# α-Lipoic Acid Antioxidant Treatment Limits Glaucoma-Related Retinal Ganglion Cell Death and Dysfunction

**DOI:** 10.1371/journal.pone.0065389

**Published:** 2013-06-05

**Authors:** Denise M. Inman, Wendi S. Lambert, David J. Calkins, Philip J. Horner

**Affiliations:** 1 Department of Neurological Surgery, University of Washington, Seattle, Washington, United States of America; 2 Department of Ophthalmology and Visual Sciences, Vanderbilt Eye Institute, Vanderbilt University, Nashville, Tennesee, United States of America; University of Regensburg, Germany

## Abstract

Oxidative stress has been implicated in neurodegenerative diseases, including glaucoma. However, due to the lack of clinically relevant models and expense of long-term testing, few studies have modeled antioxidant therapy for prevention of neurodegeneration. We investigated the contribution of oxidative stress to the pathogenesis of glaucoma in the DBA/2J mouse model of glaucoma. Similar to other neurodegenerative diseases, we observed lipid peroxidation and upregulation of oxidative stress-related mRNA and protein in DBA/2J retina. To test the role of oxidative stress in disease progression, we chose to deliver the naturally occurring, antioxidant α-lipoic acid (ALA) to DBA/2J mice in their diet. We used two paradigms for ALA delivery: an intervention paradigm in which DBA/2J mice at 6 months of age received ALA in order to intervene in glaucoma development, and a prevention paradigm in which DBA/2J mice were raised on a diet supplemented with ALA, with the goal of preventing glaucoma development. At 10 and 12 months of age (after 4 and 11 months of dietary ALA respectively), we measured changes in genes and proteins related to oxidative stress, retinal ganglion cell (RGC) number, axon transport, and axon number and integrity. Both ALA treatment paradigms showed increased antioxidant gene and protein expression, increased protection of RGCs and improved retrograde transport compared to control. Measures of lipid peroxidation, protein nitrosylation, and DNA oxidation in retina verified decreased oxidative stress in the prevention and intervention paradigms. These data demonstrate the utility of dietary therapy for reducing oxidative stress and improving RGC survival in glaucoma.

## Introduction

Age is a major risk factor for glaucoma. The free radical theory of aging promulgated the concept that oxidative stress is a key component of age-related cellular decline [1]. Reactive oxygen species (ROS), byproducts of oxidative phosphorylation, inflict oxidative insults by attacking nucleic acid, proteins and lipids. Effective management of ROS appears to decline with age [1]–[3], thereby increasing the load of dysfunctional cellular components over time. Interestingly, these oxidative insults disrupt specific proteins and pathways. For example, the α-synuclein present in Lewy bodies in Alzheimer’s and Parkinson’s diseases is specifically nitrated by oxidative stress [4]. In a rat model of glaucoma, glutamine synthetase and the heat shock protein Hsp72 were identified as targets of oxidative stress by the formation of particular protein carbonyls [5]. An analysis of optic nerve head glia and vasculature from human glaucoma patients showed significant peroxynitrite-mediated damage [6]. Further evidence of a role for oxidative stress in glaucoma is the significant serum increases in metabolites from nitric oxide production [7] and high plasma levels of the lipid peroxidation product malondialdehyde observed in glaucoma patients compared to control [8]. Glaucoma patients’ oxidative DNA damage is also significantly correlated with their visual field damage and IOP increase [9]. Decreased levels of SOD and catalase [10] concomitant with increased lipid peroxidation in retina [11] was observed in rats subjected to a model of ocular hypertension. Our studies with the DBA/2J model of glaucoma have also demonstrated increased DNA oxidation, protein nitrosylation and lipid peroxidation in the glaucomatous retina [12]. These data indicate that oxidative stress exacerbates the pathology of neurodegenerative diseases such as glaucoma.

Glaucoma patients demonstrate increased activity of antioxidant enzymes such as superoxide dismutase (SOD) and glutathione peroxidase in aqueous humor, yet show low levels of antioxidants [13]. These patients’ endogenous antioxidant response–whether through aging or pathology–is insufficient to the task of managing the oxidative stress present in glaucoma. Hence, we hypothesized that patients would likely benefit from augmentation of their antioxidant response to counter the accumulation of ocular ROS and oxidative stress. Alleviating oxidative stress has been an effective means to limit oxidative damage, providing neuroprotection [14], [15], and reducing ischemia [16], [17]. In age-related macular degeneration (AMD), antioxidant treatment slowed the progression of disease and vision loss in patients at high risk for advanced AMD [18]. α-Luminol, an antioxidant, was found to prevent the age-related decreases in glutamate, glutathione and glutamine synthetase in DBA/2J retinas [19]. Ginkgo biloba extract, which can scavenge nitric oxide, protects retinal ganglion cells from death when given to rats for 5 months after ocular hypertension [20]. Antioxidant treatment of trabecular meshwork cells *in vitro* with resveratrol led to reduced reactive oxygen species and fewer carbonylated proteins [21]. These data underscore the potential utility of reducing oxidative stress in retina and anterior chamber in glaucoma. These data prompted us to test whether increasing the antioxidant levels through dietary α-lipoic acid would relieve oxidative stress in the DBA/2J mouse model of chronic glaucoma.

The DBA/2J mouse model is one of the few glaucoma models to recapitulate the slow, progressive nature of human glaucoma. This makes the model very attractive to test potential therapies that might also be delivered to patients over decades. To date, studies that endeavor to achieve RGC neuroprotection and preservation of vision through chronic treatment of the DBA/2J mouse model have done so through lowering IOP [22], providing growth factor support [23], systemic glycoprotein hormone [24], [25] or steroid hormone [26]. Bringing these potential treatments to patients would require tremendous investment and are arguably decades away from possibility. IOP lowering, treating a risk factor for the disease, is the standard of care for glaucoma; at best, it slows the disease down. Similarly, antioxidant treatment could slow disease progression but with the added benefit of being readily available for patients now. The antioxidant α-Lipoic acid (ALA) is a naturally occurring disulfide compound synthesized in the liver and other tissues. ALA occurs naturally in the human diet; it is found in in some fruits and vegetables and in animal tissues with high metabolic activity such as heart and liver. ALA can also be synthesized *de novo* in mitochondria via lipoic acid synthase [27]. ALA, and its reduced form dihydrolipoic acid (DHLA), scavenge ROS and reactive nitrogen species [27], repair oxidized proteins and lipids, and regenerate endogenous antioxidants [28]. ALA has been used to alleviate oxidative stress in diseases such as diabetic retinopathy and neuropathy [29], [30]. ALA is currently being tested as a treatment for neurodegeneration and neuropathy in several clinical trials. A randomized, double-blind, placebo controlled trial of ALA treatment in insulin and non-insulin dependent diabetics with peripheral neuropathy showed that 1200 or 600 mg of IV ALA significantly improved sural and motor nerve conduction velocities, as well as sensory nerve action potential [29], [31]. Collectively, these data show that antioxidant treatment can slow disease progression and improve disease outcomes in clinically relevant ways [32].

In this study, we document the age and increased intraocular pressure-related oxidative stress in the DBA/2J, then use ALA in two different treatment paradigms to intervene or prevent glaucoma progression by limiting oxidative stress. As far as we are aware, this study is the only examination of chronic antioxidant delivery in the DBA2/J model of glaucoma. We show that ALA treatment is effective at increasing antioxidant levels, lowering oxidative stress-related changes in protein and nucleic acid and protecting retinal ganglion cells in the DBA/2J glaucomatous retina.

## Methods

### Ethics Statement

The University of Washington Institutional Animal Care and Use Committee approved all experimental procedures. Experiments adhered to ARVO’s statement for the use of animals in ophthalmic and visual research. All surgery was performed under appropriate anesthesia, and all efforts were made to minimize suffering.

### Animals

DBA/2J (DBA) mice were originally obtained from Jackson Labs (Bar Harbor, ME) and new DBA/2J stock was introduced into the colony periodically. All mice were bred and housed in a specific pathogen-free barrier facility at the University of Washington, Seattle. Mice were genotyped for the *Gpnmb* and *Tyrp1* mutations [33], [34]. Mice were maintained on a 12 hour light-dark cycle.

### ALA Diet

Standard rodent chow based on the American Institute of Nutrition diet 76A (AIN-76A) formulated by Research Diets, Inc. (D10001; New Brunswick, NJ) was used as the control diet; the ALA diet was supplemented with 600 mg α-lipoic acid per kg of chow (intervention study arm) or 1000 mg per kg chow (prevention study arm). Using this formula, Daily Dose = Dietary Dose (Food Intake)/Body Weight, we determined that these supplementation levels would provide 60 mg/kg body weight (bw)/day for the intervention study and 100 mg/kg bw/day for the prevention study.

We chose to supplement the mouse diet with ALA as opposed to alternative delivery routes because the half-life of racemic ALA in plasma is 30 minutes; bioavailability ranges from 20–38 percent. ALA readily crosses the blood brain barrier with peak ALA levels detected within the cortex, retina, and optic nerve within 30 minutes [27]. A no-observed-adverse-effects dose for rats was determined as 60 mg/kg bw/day [35], the dose we used in the intervention study. The 100 mg/kg bw/day used in the prevention study came about after an evaluation of the intervention study results and with the knowledge that Cremer, et al, [35], used up to 180 mg/kg bw/day for 24 months led to no observable effects of ALA on histology or pathology.

### IOP Measurement

Intraocular pressure (IOP) was measured once per month for the study mice, beginning at 2 months of age, using the TonoLab (Colonial Medical Supply, Franconia, NH) rebound tonometer. Ten IOP measurements per eye were taken before 11∶00am, under Avertin anesthesia (2,2,2-tribromoethanol (1.3%) and tert-amyl alcohol (0.8%) in dH_2_O, Sigma-Aldrich, St. Louis, MO), and the mean IOP per eye (±SEM) was calculated by averaging the ten values. For low and high IOP groups in the prevention study, mice were rank ordered according to IOP integral (mmHg-days exposure over baseline) then divided in half; the low IOP group had the lowest mmHg-days and the high IOP group had the highest mmHg-days IOP integral. There was an average difference of ≥600 mmHg-days between the low and high IOP groups (Figure S1).

### Quantitative Real-time PCR (QPCR)

Total RNA was isolated from retinas using TriZol (Invitrogen, Carlsbad, CA) and retinas from the same mouse were pooled and treated as one sample. First-strand cDNA synthesis was performed using SuperScript III First-strand Synthesis System for RT-PCR (Invitrogen, Carlsbad, CA). Primers used in this study are listed in Table 1, and when necessary were designed using mRNA sequences obtained from Entrez (www.ncbi.nlm.nih.gov) and Primer3 [36], and then verified using BLAST (www.ncbi.nlm.nih.gov). QPCR was performed in triplicate using SYBR-Green PCR Master Mix, Applied Biosystems Prism 7900 HT Sequence Detection System, and SDS2.3 software (Applied Biosystems, Foster City, CA). The Comparative CT Method (Applied Biosystems 7700 User Bulletin #2) was used to determine relative changes in gene expression levels. All other samples were normalized using the Mouse Housekeeping Gene Primer set from RealTimePrimers.com (Elkins Park, PA). Relative gene expression was determined using geNorm (http://medgen.ugent.be/~jvdesomp/genorm/ and Vandesompele J, et. al.) [37], which calculates a normalization value for each sample based on the most reliable housekeeping gene(s). Samples were normalized to at least three housekeeping genes and expression is represented graphically as mean normalized value +/- SEM.

**Table 1:** Primers used for qPCR.

Gene	Forward Primer	Reverse Primer
Ceruloplasmin	5’-TTCAACGGGCTGATGACAAAGTGC-3’	5’-GGCTTGGCCATGAAAGAAAGCTGA-3’
Glial fibrillary acidic protein	5’-CTCCGCCAAGCCAAGCACGA-3’	5’-CCAGCCGAGCAAGTGCCTCC-3’
Glutathione peroxidase 4	5’-TCTGGCAGGCACCATGTGT-3’	5’-CGGGCATGCAGATCGACTA-3’
Glutathione-S-transferase	5’- CCCAAGTTTGAGGATGGAGA-3’	5’-CAGGGCCTTCACGTAGTCAT-3’
Heme-oxygenase 1	5’-GGTCCTGAAGAAGATTGCACA-3’	5’-CTTGCACCAGGCTAGCAG-3’
Iba1	5’-CCTGATTGGAGGTGGATGTCAC-3’	5’-GGCTCACGACTGTTTCTTTTTTCC-3’
Lipocalin 2	5’-ACAATGTCACCTCCATCCTGGTCA-3’	5’-CAAAGCGGGTGAAACGTTCCTTCA-3’
Nitric oxide synthase-2	5’-TCATTGTACTCTGAGGGCTGACACA-3’	5’-GCCTTCAACACCAAGGTTGTCTGCA-3’
Nrf2	5’-TGAAGCTCAGCTCGCATTGATCC-3’	5’-AAGATACAAGGTGTCTGAGCCGCC-3’

### Protein Extraction and Western Blot Analysis

Cellular protein was collected from retinas removed from freshly enucleated eyes. Retinas from the same mouse were pooled and cell lysate (20 µg) was separated on denaturing polyacrylamide gels and transferred by electrophoresis to PVDF membranes. The following antibodies were used for Western blotting: GFAP (Dako North America, Inc., Carpinteria, CA), heme oxygenase-1 (Stressgen Bioreagents Corp., Victoria B.C.), nitric oxide synthase-2 (Chemicon International Inc., Temecula, CA), and RAGE (Santa Cruz Biotechnology, Santa Cruz, CA). Blots processed using primary antibodies (diluted 1∶1000 in Tris buffered saline (TBS) with 0.2% Tween-20 and 1% milk or BSA) were then exposed to secondary antibodies conjugated to horseradish peroxidase (diluted 1∶10,000; Jackson ImmunoResearch Laboratories Inc., West Grove, PA), developed using chemiluminescence and exposed under Kodak X-ray film for various times depending on the amount of target protein present. Band density was measured using ImageJ version 1.38× (http://rsb.info.nih.gov/ij/index.html) and was normalized to β-actin (diluted 1∶5000; Abcam, Inc., Cambridge, MA). Expression is represented graphically as mean normalized value +/- SEM.

### Immunohistochemistry

Mice euthanized with 300 mg/kg sodium pentobarbital were perfused with 4% paraformaldehyde (PFA) in 0.1 M phosphate buffer. Eyes and attached optic nerves were dissected from the skull, and the anterior segment was separated from the posterior eyecup, which was then post-fixed for 1 h in 4% PFA. For sectioning, eyecups were rinsed in phosphate buffer (PB) (pH 7.4) and transferred to 30% sucrose in PB overnight. Eyecups were then embedded in OCT Tissue-Tek (Sakura Finetek; Torrance, CA) and frozen in liquid nitrogen for sagittal sectioning on a Leica cryostat. For wholemount staining, retinas were dissected from the eyecup and, following vitreous removal, were cryoprotected in 30% sucrose. The following antibodies were used for immunohistochemistry: 3-nitrotyrosine (1∶200; Upstate, Lake Placid, NY), 8-hydroxyguanosine/8-hydroxy-deoxyguanosine (1∶250; QED Bioscience Inc, San Diego, CA), βIII-tubulin (1∶500; Covance, Princeton, NJ), Fluorogold (1∶500; Chemicon International Inc., Temecula, CA), GFAP (1∶500; Advanced ImmunoChemical Inc., Long Beach, CA), GLAST (1∶250; Chemicon International Inc., Temecula, CA), NeuN (1∶500; Chemicon International Inc.), neurofilament-heavy (1∶500; Sigma-Aldrich, St. Louis, MO), neurofilament-light (1∶250; Chemicon International Inc). For immunohistochemistry, sections and wholemounts were blocked for 1 hour and incubated overnight in primary antibodies. Tissue was rinsed, blocked for 30 min, and then incubated for 2 hours in appropriate secondary antibodies (1∶500; Jackson ImmunoResearch Laboratories Inc., West Grove, PA). After rinsing, slides were mounted in Gelvitol. Images were captured using a BioRad Radiance 3-channel confocal microsope and 3D image stacks created for analysis in Volocity (Improvision Inc., Lexington MA).

### Retrograde Labeling of RGCs

Anesthetized (Avertin) mouse scalps were shaved, swabbed with Betadine, and placed in a Kopf stereotaxic device (David Kopf Instruments, Tujunga, CA, USA) with mouse adaptor. Following a midline incision in the scalp, bilateral holes were drilled at –4.0 Bregma, ± 0.5 midline and 1 µL per site of 1% FluoroGold (FG; hydroxystilbamidine, methanesulfonate; Invitrogen, Carlsbad, CA) in dH_2_O was injected via Hamilton syringe 0.5 mm below dura into the superior colliculus. Injections were made over 2 minutes and removal of syringe was made over 1 minute. Gelfoam pledgettes soaked in 5% FG were placed within each drill hole on the surface of the colliculus. Scalps were then sutured and mice placed on a warm water blanket until recovery from anesthesia. Tissue was collected as described 7 days following injection.

### RGC Quantification

Unbiased stereological analysis of NeuN+ and FluoroGold+ cell numbers was performed in whole mount retinas as previously described [38] using the optical fractionator module within StereoInvestigator (MicroBrightfield, Middlebury, VT). A 50 µm×50 µm counting frame was utilized within a sampling grid incorporating the entire retina; an average of 50 random sampling sites per retina were examined and cells counted to obtain a representation of NeuN+ and FluoroGold+ cells per mm^2^ within each whole mount retina. Criteria for counting a NeuN-positive retinal ganglion cell included a minimal somal size of 10 µm and a prominent nucleolus, criteria that eliminated the vast majority of displaced amacrine cells from the NeuN count. Nevertheless, NeuN counts were corrected for amacrine cells using a previously published [38] correction factor that indicated 11.4 percent of NeuN-positive cells with greater than 10 µm somal size in 10 to 12-month-old retinas are displaced amacrine cells.

### Lipid Peroxidation Assay

Lipid peroxidation was assayed using the Lipid Peroxidation Assay Kit (Oxford Biomedical Research, Oxford MI), a colorimetric assay that measures the reaction between a chromogenic reagent R1 and malondialdehyde (MDA), the end product from peroxidation of polyunsaturated fats. Mice euthanized with 300 mg/kg sodium pentobarbital were perfused with a 0.9% saline solution, the eyes were removed, and the retinas rinsed in ice-cold saline, weighed, and then homogenized in Tris buffer (pH 7.4) containing BHT to prevent sample oxidation. Following centrifugation supernatant is mixed with R1 and HCl, incubated at 45°C, cooled on ice and the absorbance at 586 nm measured. A standard curve was generated and the concentration of MDA in the sample expressed as nmoles MDA per mg retinal protein ± SEM.

### Glutathione Assay

Whole retina was prepared according to the Glutathione Assay Kit instructions (703002, Cayman Chemical, Ann Arbor, Michigan) Glutathione in the sample reacts with Ellman’s reagent, producing a disulfide that is reduced by glutathione reductase. Measurement of absorbance of the disulfide at 405 nm provides an accurate estimate of total glutathione (both in reduced GSH and oxidized GSSG form).

### Optic Nerve Analysis

A 3-mm section of optic nerve proximal to the globe was isolated, post-fixed for 1 hour in 4% PFA, and prepared for embedding and semi-thin sectioning, as previously described [39]. Using 100× oil-immersion and differential interference contrast optics, photomicrographs of each section were collected as a montage with a microscope equipped with a motorized X-Y-Z stage and a digital charge-coupled device video camera (Provis AX70; Olympus). Each montage was contrast- and edge-enhanced using macro-routines written in ImagePro (Media Cybernetics, Silver Spring, MD). An additional routine was used to identify, count and determine axon area for each axon in the montage for which a myelin sheath could be identified. We used this information to calculate the mean local axon density for each optic nerve.

For optic nerve grading, we used the methods described in Chauhan, et al., in which nerve cross-sections are divided into regions with similar levels of degeneration [40]. Regions are evaluated for the level of degeneration, ranging from 0 (no degeneration) to 10 (100 percent degeneration). The percent total area of a region is used to weight the level of degeneration and these weighted levels are summed to provide an integer value for degeneration level [40]. Image J was used to delineate degeneration regions in high-magnification montages of optic nerve. A region’s area was converted to percentage area of optic nerve then multiplied by the percentage of degeneration in that region. These values were summed then multiplied by 10 to yield grade which corresponds to degeneration level. Three independent, blinded observers graded the optic nerves.

### Visual Cortex c-fos Labeling

Mice were housed in a 12∶12 light-dark cycle, and the experiment took place from Zeitgeber (ZT)13 to ZT16. Dark-adapted mice were placed in a 8.5-inch square box with black sides (control) or vertical striped sides (black and white stripes, 0.5-inches wide). An overhead 450 lux white light was turned on for 10 min while mice were in their respective boxes. Mice were returned to their home cages and kept in the dark, then perfused 80 minutes later. Mice were euthanized with 300 mg/kg sodium pentobarbital then perfused transcardially with 0.1 M PB followed by 4% PFA. Brains were removed, cryoprotected, then sectioned and V1 visual cortex was immunolabeled with antibodies against c-fos.

Brain sections were incubated overnight in primary antibody (Calbiochem Ab-5; 1∶20,000 in 0.1 M Tris buffer with 5% donkey serum and 0.1% Triton X-100) and developed with a Vectastain HRP kit (Vector Labs, Burlingame, CA) and DAB (Metal Enhanced DAB substrate kit, Thermo Scientific, Rockford, Il). Sections were mounted on microscope slides, dehydrated in graded alcohols to xylenes, then coverslipped with Permount.

### Statistical Analysis

Unless otherwise indicated, all data are presented as the mean ± the standard error of the mean (SEM). Prism 5 (GraphPad Software; San Diego, CA) was used to calculate correlation coefficients and corresponding p values. Groups were compared using a One-Way ANOVA followed by a Tukey’s Multiple Comparison post-test. If variances were statistically different, data was then analyzed using a Kruskal-Wallis test followed by a Dunn’s Multiple Comparison post-test. Minimum significance was set at p < 0.05. Control and ALA treated groups were compared using an unpaired t-test; if variances differed significantly, data was then analyzed using an unpaired t-test with Welch’s correction. Minimum significance was set at p < 0.05.

## Results

### Oxidative Stress in the DBA/2J

**Figure 1:**
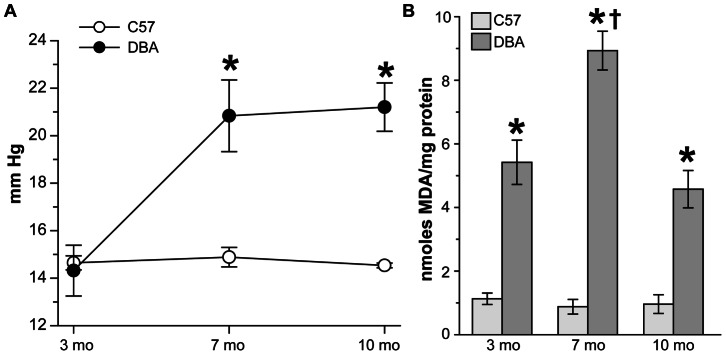
Lipid peroxidation increases in DBA retinas with age and IOP. **A**. Ocular pressure in mmHg was measured in C57 and DBA mice prior to lipid peroxidation assay (n = 6). IOP did not change with age in C57 mice, ranging from 14.8 mmHg at 3 months to 14.5 mmHg at 10 months (p>0.07). In DBA mice IOP increased 6.8 mm Hg (48%) with age (*p<0.001) resulting in pressure elevations of 40 and 46% in DBA mice at 7 and 10 months of age compared to C57 mice (* p<0.001). **B**. Bar chart comparing nmoles of malondialdehyde (MDA) per mg protein in retinal lysate from C57 and DBA mice (n = 6). Levels of MDA were 480% higher in 3 month and 10 month DBA retinas and 1017% higher in 7 month DBA retinas compared to age-matched C57 mice (*p<0.0001). MDA levels did not change in C57 retinas with age (p>0.71) but were 180% higher in 7 month DBA retinas compared to 3 and 10 month DBA mice († p<0.001).

To determine if DBA/2J (hereafter DBA) retinas undergo oxidative stress with age and increasing intraocular pressure, we measured IOP and the levels of malondialdehyde (MDA), a common by-product of lipid peroxidation (Figure 1). At three months of age, IOP was similar in C57 (14.6 ± 0.3 mm Hg) and DBA mice (14.3 ± 1.1 mm Hg) (Figure 1A; p = 0.48). Pressure did not change significantly in C57 mice with age (14.8 ± 0.4 mm Hg at 7 months, and 14.5 ± 0.1 mm Hg at 10 months; p>0.07), but did increase in DBA mice (47% on average, *p<0.001) to 20.8 ± 1.5 mm Hg at 7 months, and 21.2 ± 1.0 mm Hg by 10 months of age. Lipid peroxidation levels (Figure 1B) in C57 retinas did not change with age (p>0.71), averaging 0.99 ± 0.1 nmoles MDA/mg protein (range of 0.87 to 1.12 nmoles MDA/mg protein). In contrast, MDA levels increased 180% in 7 month DBA retinas († p<0.001), reaching a peak of 8.93 ± 0.6 nmoles MDA/mg protein, compared to 3 and 10 month DBA retinas which had similar MDA levels (4.8 ± 0.6 vs. 4.7 ± 0.5 nmoles MDA/mg protein; p = 0.22). As a result, MDA levels were significantly higher in DBA retinas at every age examined compared to age-matched C57 retinas (*p<0.0001).

### Increased Expression of Oxidative Stress-related Genes and Protein in DBA

**Figure 2:**
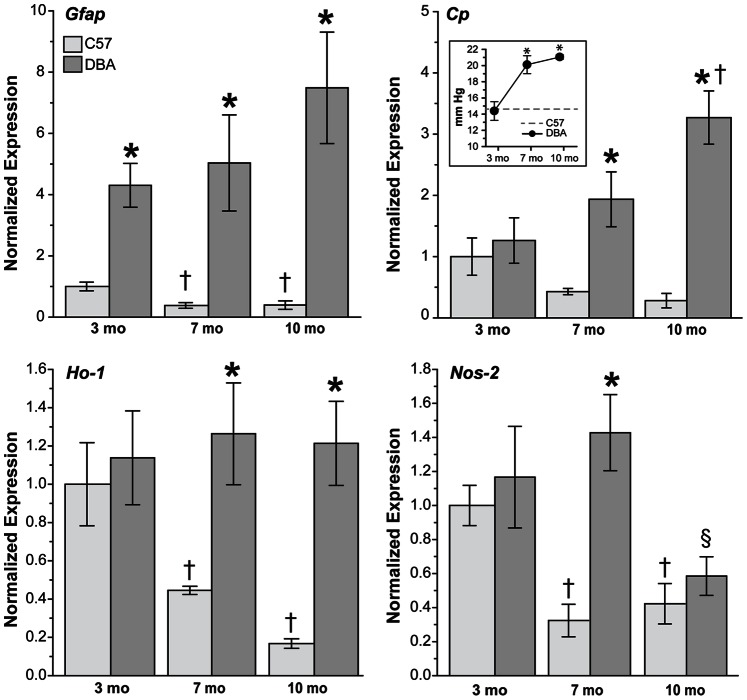
Upregulation of oxidative stress-related genes in DBA retinas with age and IOP. Bar charts demonstrating oxidative stress-related gene expression in C57 and DBA retinas by qPCR. Gfap decreased 40% in C57 retinas (p>0.67) and increased 47% in DBA retinas (p>0.10) with age. Overall Gfap levels were increased 330% to 1812% in DBA retinas when compared to age-matched C57 retinas (*p<0.001). Cp decreased with age in C57 mice (64.5%; p>0.12) while increasing 183% in DBA mice († p<0.001), which resulted in increased Cp expression in DBA retinas at 7 months (422%) and 10 months (1172%) compared to age-matched C57 mice (*p<0.004). Ho-1 did not change in DBA retinas with age (p>0.65), but decreased 67% in C57 mice (†p>0.009). Compared to C57 retinas, Ho-1 levels increased 184% at 7 months and 625% at 10 months of age in DBA retinas (*p<0.001). Nos-2 decreased with age in C57 retinas (63%; †p<0.003) and increased DBA retinas at 7 months (22%; p>0.2) prior to decreasing at 10 months (59%; §p>0.003). Nos-2 levels were similar in C57 and DBA retinas at 3 months (p = 0.51) and 10 months (p = 0.52), but were increased 341% at 7 months of age (*p<0.001). (Inset) IOP of C57 (average over all ages) and DBA mice used for qPCR (n = 5). Intraocular pressure increased 52% on average in DBA mice with age (*p<0.001). IOP was lower (8%) in 3 month DBA mice compared to C57 mice (p = 0.016); however IOP was elevated 37% by 7 months of age and 44% by 10 months of age in DBA mice compared to C57 mice (*p<0.001).

**Figure 3:**
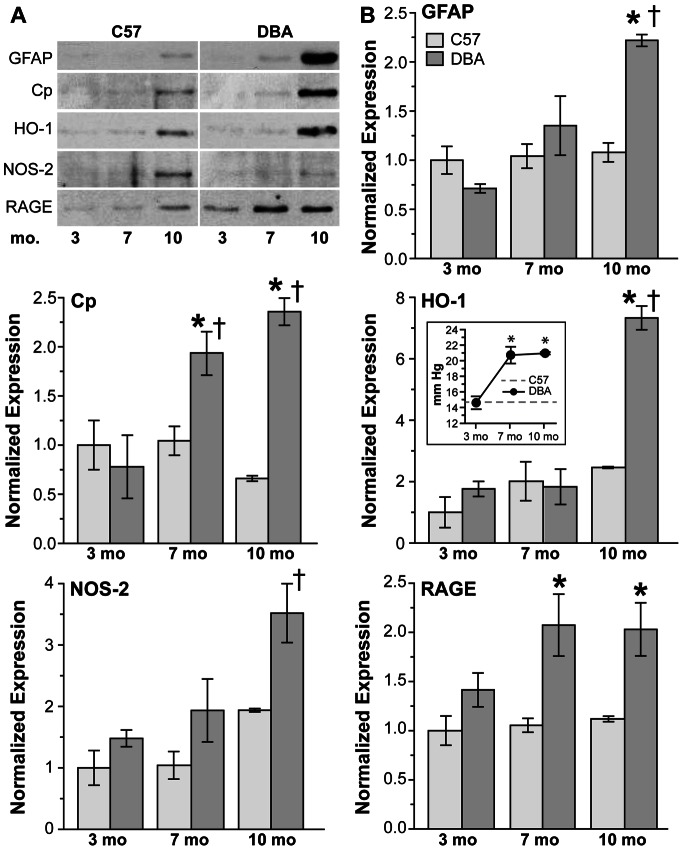
Upregulation of oxidative stress-related proteins in DBA retinas with age and IOP. **A**. Representative western blots and **B**. bar charts demonstrating oxidative stress-related protein expression in C57 and DBA retinas. GFAP did not change with age in C57 retinas (p>0.72), and did not increase significantly in DBA retinas until 10 months of age (211%; †p = 0.003), resulting in a 106% increase in GFAP in DBA retinas at this age (*p<0.001). With age Cp decreased in C57 retinas (36%; p>0.21) and increased in DBA retinas (147% to 203%; †p<0.007); as a result Cp levels in DBA retinas were increased 84% and 257% at 7 and 10 months of age, respectively, compared to C57 retinas (*p<0.001). HO-1 increased with age in both C57 (124% on average; p>0.13) and DBA retinas (316%; †p<0.001), resulting in levels 198% higher in 10 month DBA retinas compared to 10 month C57 retinas (*p<0.001). NOS-2 increased 94% in C57 (p>0.06) and 138% DBA retinas (†p = 0.03) with age. NOS-2 levels were increased in DBA retinas compared to C57 retinas at all ages examined (72% on average; p>0.07). RAGE did not change in C57 retinas (p>0.67) but did increase 44.5% in DBA retinas with age (p>0.214). As a result, RAGE increased 97% and 81% at 7 and 10 months compared to C57 retinas (p<0.006). (Inset) IOP of C57 (average over all ages) and DBA mice used for western blotting (n = 3). IOP was similar in C57 and DBA mice at 3 months of age, but due to a 48% increase in DBA IOP with age, was 42.5% higher on average in DBA mice compared to age-matched C57 mice (*p<0.001).

In addition to lipid peroxidation, oxidative stress-related genes were upregulated in DBA retinas with increasing age and IOP (Figures 2 and 3). IOP values for C57 and DBA mice used for qPCR (Figure 2 inset) and western blotting (Figure 3 inset) showed that pressure did not increase significantly with age in C57 mice (p>0.08); therefore, IOP readings were averaged over all ages of C57 examined and shown as a dotted gray line in both graphs. As expected, IOP increased with age in DBA mice used for qPCR (Figure 2 inset) from 14.4 ± 1.1 mm Hg at 3 months of age to 20.2 ± 1.1 mm Hg and 21.1 ± 0.3 mm Hg at 7 and 10 months of age, respectively (*p<0.001). IOP was similar in 3 month DBA mice (p = 0.74) before becoming significantly elevated (37 to 44%) by 7 and 10 months of age (*p<0.001). We used GFAP as a marker for retinal stress as its expression is upregulated in astrocytes and Müller glia during reactive gliosis, and because expression increases significantly in DBA mice with age [41]. In C57 retinas, *Gfap* levels decreased 60% with age (Figure 2; †p = 0.005), while in DBA retinas, *Gfap* increased 17% at 7 months and 74% at 10 months (p>0.10). Ceruloplasmin (*Cp)* expression was decreased in C57 retinas (65%; p>0.12) and increased 77% to 183% in DBA retinas with age (†p<0.001). While similar in 3-month C57 and DBA mice (p = 0.56), *Cp* increased 422% in DBA retinas at 7 months and 1172% at 10 months compared to age-matched C57 mice (*p<0.004). Ceruloplasmin is a copper oxidase that can scavenge free radicals and oxidize ferrous iron [42]. Heme oxygenase-1 (*Ho-1*) expression can be induced by various triggers including oxidative stress [43]–[45], and while *Ho-1* remained constant in DBA retinas with increasing age (p>0.65), mRNA levels decreased 55% to 83% in C57 retinas with age (†p<0.009). As a result, *Ho-1* expression in DBA retinas increased 184% at 7 months and 625% at 10 months (*p<0.001). The inducible form of nitric oxide synthase, *Nos-2*, decreased 63% on average with age in C57 retinas (†p>0.003). In DBA retinas, *Nos-2* increased 22% at 7 months of age and then decreased 59% at 10 months (§p = 0.003). While *Nos-2* levels were similar in 3 month C57 and DBA retinas (p = 0.51) and in 10 month C57 and DBA retinas (p = 0.52), a 341% increase was observed in DBA retinas at 7 months of age compared to C57 mice (*p<0.001).

Oxidative stress-related protein expression was also upregulated in DBA retinas as age and IOP increased (Figure 3). While not different from C57 mice (14.6 ± 0.7 mm Hg; p = 0.87) at 3 months of age, IOPs were elevated 42.5% in DBA mice at 7 and 10 months of age (Figure 3 inset; *p<0.001). Age did not affect GFAP levels in C57 retinas (p>0.72), while a 211% increase was observed in 10 month DBA retinas (†p = 0.003), which resulted in a 106% increase in GFAP in DBA retinas at this age (*p<0.001). CP decreased slightly with age in C57 retinas (p>0.21), but increased significantly in 7 and 10 month DBA retinas respectively (†p<0.007). Although HO-1 was upregulated with age in both C57 (124%; p>0.13) and DBA retinas (316%; †p<0.001), levels were similar between the two strains (p>0.2) until 10 months of age, when HO-1 increased 198% in DBA retinas compared to age-matched C57 retinas (*p<0.001). NOS-2 also increased with age in C57 (94%; p>0.06) and DBA retinas (138%; †p = 0.03), and while NOS-2 was increased in DBA retinas compared to C57 retinas at all ages examined (72% on average) this result was not significant (p>0.07). Advanced glycation end products (AGEs) covalently cross-link proteins to form aggregates and can, through their cell surface receptor RAGE, lower endogenous antioxidant levels [46], [47]. RAGE levels were similar in C57 retinas across all ages (p>0.67), but did increase in DBA retinas at 7 and 10 months of age (p>0.214).

**Figure 4:**
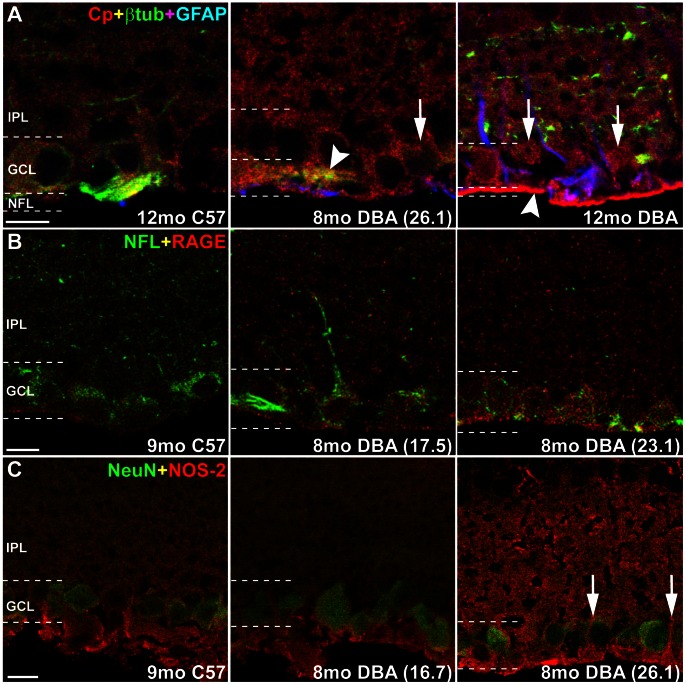
Cp, RAGE and NOS-2 increase in DBA retinas with age and IOP. Representative sagittal sections of C57 and DBA eyes immunolabeled for oxidative stress proteins. Terminal IOP for DBA mice in parentheses. All photomicrographs are taken from the mid-central portion of the retina in a section that contains the optic nerve; n = 3. **A**. Cp (red) labeling was faint in the inner plexiform layer (IPL), ganglion cell layer (GCL) and nerve fiber layer (NFL) in C57 retinas. Cp colocalized with βIII-tubulin (green) in RGC somas and axons in the GCL and NFL, and with GFAP-positive astrocytes (blue) in the NFL. In DBA retinas, Cp increased in the IPL and in RGC somas (arrows) and axons (arrowheads) as age and IOP increased. **B**. RAGE (red) labeling in C57 retinas was faint and concentrated in the GCL. Intensity of RAGE labeling increased in DBA retinas and colocalized with neurofilament-light (NFL; green) in RGCs. **C**. NOS-2 (red) labeling was primarily located in the GCL and NFL in C57 retinas, and did not colocalize with NeuN (green), a marker for RGCs and amacrine cells. Similar NOS-2 localization and intensity was observed in retinas from DBA mice with low IOP (<18 mmHg). Labeling appeared to be more intense in retinas from DBA mice with high IOP (>21 mmHg), especially in the IPL and what appeared to be Müller cell processes in the NFL and GCL (arrows). Scale: 10 µm.

To localize expression of oxidative stress-related proteins within the retina, we performed immunochemistry using antibodies to CP, RAGE and NOS-2 along with cell-specific markers in sagittal sections of C57 and DBA retina. CP was observed as faint labeling in the IPL, GCL and NFL in C57 retina (Figure 4A). In DBA retina, CP colocalized with βIII-tubulin in RGC somas and axons in the GCL and NFL, and with GFAP-positive astrocytes in the NFL. In DBA retinas, CP increased in the IPL and in RGC somas and axons as age and IOP increased (Figure 4A, right). RAGE immunolabeling in C57 retinas was faint and concentrated in the GCL. Intensity of RAGE labeling increased in DBA retinas and colocalized with neurofilament-light in RGCs (Figure 4B). NOS-2 labeling was primarily located in the GCL and NFL in C57 retinas, and did not colocalize with NeuN, a marker for neurons that labels RGCs and amacrine cells in GCL. Similar NOS-2 localization and intensity was observed in retinas from DBA mice with low IOP (<18 mmHg). Labeling appeared to be more intense in retinas from DBA mice with high IOP (>21 mmHg), especially in the IPL and what appeared to be Müller glia processes in the NFL and GCL (Figure 4C, right).

### Protein Nitration and DNA Oxidation Increase in DBA Retinas

**Figure 5:**
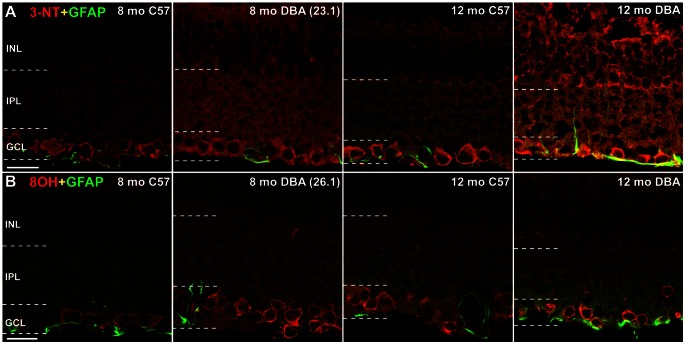
Protein nitration and DNA oxidation increase in DBA retinas with age and IOP. **A**. Comparing 8 and 12 month C57 to DBA sectioned retina illustrates the glaucoma-related increase in protein nitration (3-NT immunolabeling) in the INL, IPL and GCL of DBA, including in astrocyte processes (12 month DBA colocalization of 3-NT and GFAP). Terminal IOP in parentheses; n = 3. Scale: 20 µm. **B**. Immunolabel for 8OHdG, a marker of nucleic acid oxidation, is strong in the GCL of 8 and 12-month-old DBA retina. Scale: 20 µm.

Protein nitration due to oxidative stress occurs when nitric oxide and oxygen radicals combine to form peroxynitrite, which leaves a “footprint” that can be detected using antibodies to 3-nitrotyrosine (3-NT) [48]. Figure 5A shows 3-NT immunolabeling concentrated in the GCL for both C57 and DBA retina, though the 3-NT is observed throughout the retina and at higher labeling intensity in DBA. In the 12-month DBA retina, the 3-NT immunolabel is colocalized with GFAP, demonstrating protein nitration in glial cells. As peroxynitrite is not the sole source of tyrosine nitration, we also examined myeloperoxidase [49] and found little to no expression in DBA retinas by immunocytochemistry (data not shown). Oxidation of DNA and RNA results in the modified nucleotides 8-hydroxy-deoxyguanosine (8OHdG) and 8-hydroxy-guanosine (8OHG) respectively. We used an antibody that recognizes both 8OHdG and 8OHG to determine if RNA and DNA oxidation occurs in DBA retinas (Figure 5B). Both 8 and 12-month retina from C57 and DBA mice are shown. There is higher density of the 8OHdG immunolabel in the GCL of DBA compared to the C57, including some colocalization with GFAP in the 12-month DBA.

### ALA does not Alter IOP Timecourse

Our data indicates oxidative stress mechanisms at work in the DBA retina in the course of glaucoma development. We used these data to design an intervention study in which we would deliver an antioxidant, α-lipoic acid, orally to DBA mice beginning at 6 months of age in an effort to intervene in the oxidative stress mechanisms and alter the progression of glaucoma. DBA mice were provided with ALA in their chow at a dose of ≥60 mg/kg of body weight (bw)/day (see Methods for details).

**Figure 6:**
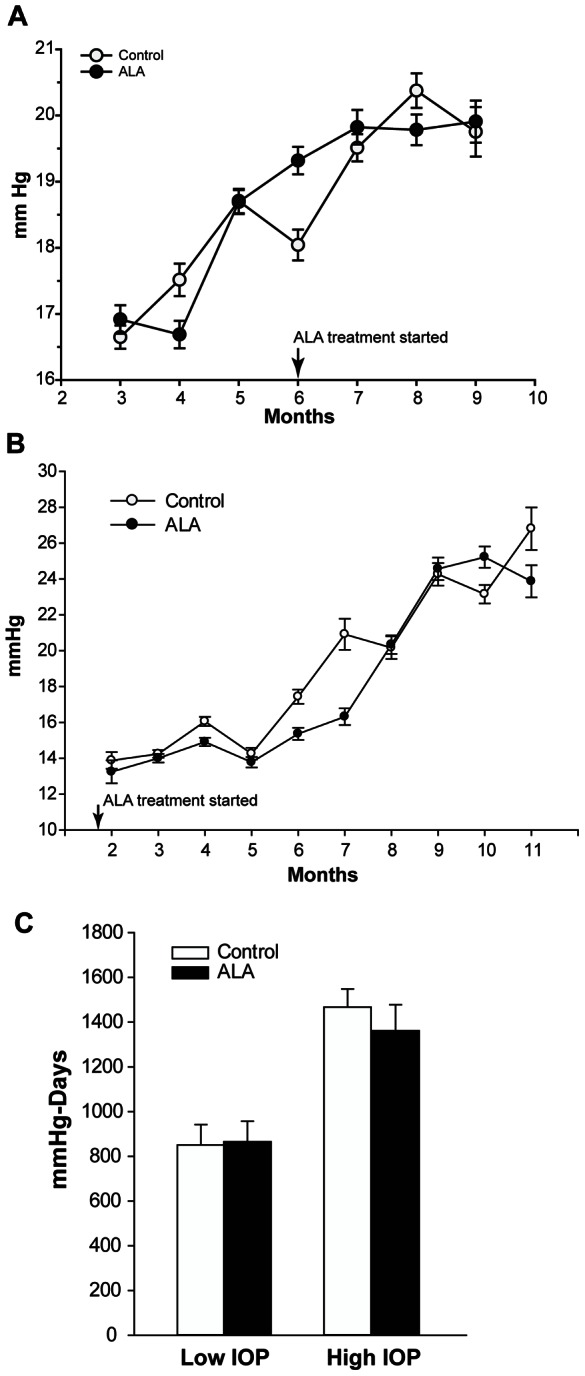
ALA does not affect IOP in DBA mice. **A.** *Intervention Study*: Treatment with ALA began at 6 months of age and continued for four months. Non-treated control (solid circles; n = 30) and ALA treated (open circles; n = 29) DBA/2J mice had similar average IOPs before and during treatment. **B.** *Prevention study:* Treatment with ALA began at weaning, 21 days, and continued until mice were 12 months of age. IOP was measured once per month beginning at 2 months. Control (n = 96) and ALA (n = 96) treated DBA/2J mice had similar average IOPs throughout treatment. **C**. For the prevention experiment, IOP exposure, expressed as mmHg-Days, was calculated by multiplying the measured IOP by the number of days at that IOP and then summing the values. There was no difference in IOP exposure between control and ALA treatments within the low or high IOP category, but the average difference across low and high IOP categories (≥600 mmHg-Days) is significant (*p = 0.001). All mRNA and protein analysis for the prevention experiment was done by IOP level in the control and ALA treatment groups, but was only presented by IOP level when the results were statistically different.

ALA dosing did not affect IOP in the intervention study (Figure 6A). Both control and ALA groups ended the study at 10 months of age with similar IOP (20.2 and 19.9 mmHg, respectively). As expected for DBA mice, these IOPs were significantly increased over the measures taken at 3 months of age (p<0.001). ALA in the chow did not negatively affect food intake or normal weight gain (Table 2).

**Table 2:** Intervention Study Outcomes.

	Control	ALA
Final Lifetime IOP (mmHg)	20.2±0.39	19.9±0.28
Average Daily Food Intake (g)	3.26±0.28	3.08±0.23
Average Weight Gain (g)	0.03±0.005	0.02±0.005
Average Daily Dose ALA (mg/kg)	0	64.15

In addition to the intervention study, we tested whether ALA treatment could prevent the RGC loss associated with glaucoma by placing DBA/2J mice on an ALA or control diet immediately after weaning and following them until 12 months of age. The daily ALA dose was increased to ≥100 mg/kg per day. Mice had their IOP taken once per month during the study. ALA treatment from weaning did not significantly alter IOP (Table 3 and Figure 6B) compared to control DBA mice. Both groups had significant IOP increase over measures taken at 2 months of age (p<0.001). In addition, both groups had comparable daily food intake and weight gain over the 12 months of the experiment (Table 3).

**Table 3:** Prevention Study Outcomes.

	Control	ALA
Final Lifetime IOP (mmHg)	26.8±5.93	23.87±4.19
Average Daily Food Intake (g)	2.78±0.28	2.81±0.25
Average Weight Gain (g)	7.11±2.89	6.58±1.39
Average Daily Dose ALA (mg/kg)	0	107.53±8.27

### ALA Intervention Treatment Enhances Innate Antioxidant Capacity

**Figure 7:**
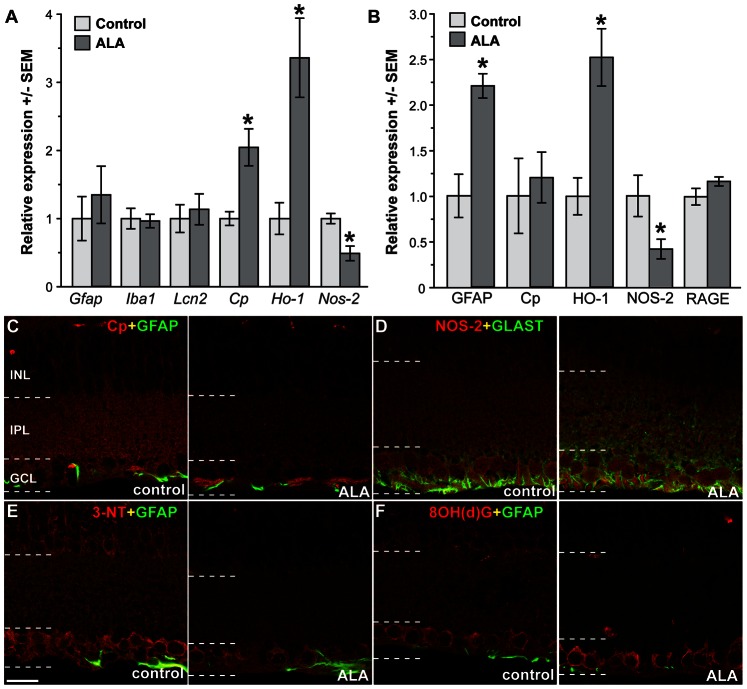
Dietary ALA treatment of DBA/2J mice (intervention experiment) altered expression of key mRNA and protein. **A**. Expression of stress-related mRNAs in ALA treated and non-treated DBA mice by QPCR. * p = 0.02 for Cp, p = 0.04 for Ho-1, and p = 0.004 for Nos-2 versus control (n = 5 per group). **B**. Expression of stress-related genes in ALA treated and non-treated DBA mice by western blot. Representative western blots are shown for non-treated and ALA treated DBA/2J retinal samples. *p = 0.04 for Nos-2, p = 0.04 for Ho-1, and p = 0.004 for Gfap versus control (n = 4 per group). **C**. CP immunolabel (red) was more concentrated in the GCL-NFL of the ALA treated retina compared to control. **D**. NOS-2 (red) showed little change in control versus ALA. **E**. 3NT immunoreactivity appeared more intense in the INL and in RGCs within the GCL of control DBA retinas when compared to ALA treated retinas. 3NT colocalized with GFAP in astrocytes (arrows). **F**. Control and ALA treated DBA retinas demonstrated 8OH(d)G immunoreactivity within RGCs in the GCL. 8OH(d)G was also observed in the INL in control retinas. Scale: 20 µm.

Dietary ALA resulted in significant changes in oxidative stress-related mRNA and protein (Figure 7). Retinas from ALA-treated mice showed significant increases in *Cp* (*p = 0.02) and *Ho-1* (*p = 0.04) mRNA and a significant decrease in *Nos-2* (*p = 0.004). There were no changes in *Gfap, Iba1* and *Lcn2* mRNA levels with ALA treatment. Using Western blot to quantify protein, we observed no changes in CP and RAGE levels in ALA versus control treatment, though there was significantly more GFAP protein in the ALA-treated group compared to control (*p = 0.004). Significant differences in *Ho-1* and *Nos-2* mRNA levels were sustained at the protein level for HO-1 (increased in ALA) and NOS-2 (decreased in ALA) (Figure 7B). Results indicate that ALA treated mice displayed mRNA and protein changes consistent with enhanced antioxidant mechanisms. Compared to DBA baseline measures of these transcripts, ALA treatment enhanced *Ho-1* expression, lowered *Nos-2* and *Gfap* expression and led to slightly lower *Cp* mRNA levels (2-fold increase versus 4-fold increase in DBA) (Figures 2 and 7A). Protein levels of RAGE, NOS-2, CP and HO-1 were all slightly lower in ALA treated DBA compared to DBA baseline, while GFAP maintained its raised protein levels throughout ALA treatment (Figures 3 and 7B).

Immunolabeling ALA and control retinas for CP or NOS-2 and markers for glial cells, GFAP and GLAST, showed an increase in the GCL for CP expression and concomitant CP decrease in IPL and INL in the ALA treated retina; there was little change in NOS-2 protein distribution and intensity (Figure 7C–D). CP and NOS-2 are increased with age and IOP in the DBA (Figures 3 and 4), while the immunolabeling in Figure 7 indicates that ALA treatment slightly decreased CP and NOS-2 expression overall (Figure 7C–D). 3-NT, the marker of protein nitration, was decreased in the ALA treated retina, as was the immunolabel for 8OHdG, suggesting that ALA treatment was effective at reducing the oxidative stress-related damage to protein and nucleic acid (Figure 7E–F). Compare these results to Figure 5 which shows the increase in DBA 3-NT and 8OHdG immunolabel with age and increasing IOP.

### a-Lipoic Acid Intervention Preserves RGC Soma and Axon

**Figure 8:**
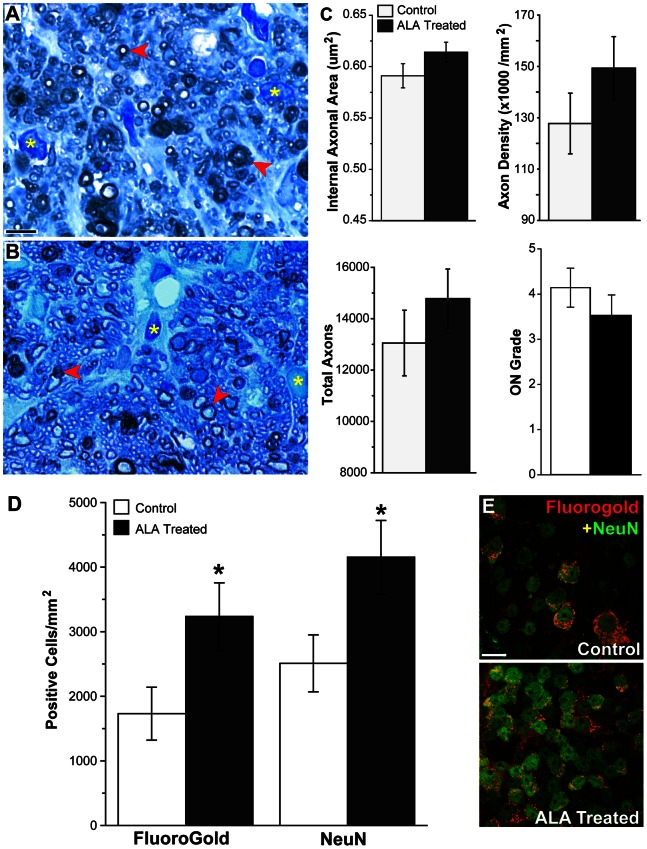
Dietary ALA treatment of DBA mice (intervention experiment) preserves RGC axons and cell bodies. **A** and **B**. Representative images showing axon degeneration (red arrows) and gliosis (yellow*) are present in *p*-phenylenediamine (PPD) stained optic nerves from control and ALA treated DBA mice. Fewer degenerating profiles are observed in ALA treated DBA optic nerves. Scale: 10 µm. **C**. Axon area, axon density, total axon number and ON grade from control and ALA treated optic nerve. There were no significant differences between control (n = 32) and ALA (n = 32) groups for these indices (ON grade, p = 0.16). See Methods for description of ON grading scheme; 0 = no degeneration while 10 = 100% degeneration. **D**. Unbiased stereology was used to count FluoroGold-positive and NeuN-positive cells in control and ALA treated DBA whole mount retinas; n = 15 per group. These counts have been corrected for displaced amacrine cells (see Methods). The number of FG-positive cells in control retinas was 8046 ± 1915 cells compared to 21408 ± 4492 cells in ALA treated retinas (p<0.021). The number of NeuN-positive cells in control retinas was 19062 ± 3146 cells compared to 36048 ± 5128 cells in ALA treated retinas (p<0.018). **E**. Representative images from control and ALA treated whole mount retinas showing NeuN (green) labeled and FluoroGold (red) labeled cells. Scale: 10 µm.

ALA treatment had an identifiable, though not statistically significant, preservation effect on the number of RGC axons in the optic nerve (Figure 8). Representative optic nerve cross-sections show greater astrocyte hypertrophy and fewer intact myelinated axons in the control as compared to the ALA treatment group (Figure 8A, B). There were 13 percent more axons in the ALA treated optic nerves; they were also at slightly higher density and had 4 percent more surface area. The optic nerve degeneration grade (ON Grade) was also lower for the ALA group (Figure 8C). ON grade is calculated by summing the weighted average of the percentage of degeneration in the nerve (see Methods). Lower ON grade corresponds to lower percentage of degeneration. ALA treatment led to preservation of RGCs in the retina (Figure 8D). Control and ALA-treated mice were given injections of FluoroGold in the superior colliculus to label RGCs with intact connections and retrograde transport systems. Retinas were also labeled for NeuN, a marker of neuronal cell bodies. There was significantly higher NeuN+ and FG+ cell density in the GCL of ALA treated mice (p = 0.045, p = 0.047, respectively), suggesting RGC structural and functional protection with dietary ALA. The NeuN numbers are corrected for displaced amacrine cells using a previously determined correction factor [38] and by excluding neurons in the GCL that are NeuN-positive but do not have a prominent nucleolus nor somal size greater than 10 µm. In addition, immunolabeling for βIII-tubulin shows many more RGCs in the ALA treatment group with strong βIII-tubulin throughout their somas and fasciculated axons compared to control retina (Figure S2).

### Prevention Study

#### Comparison of Prevention and Intervention Study outcomes

**Figure 9:**
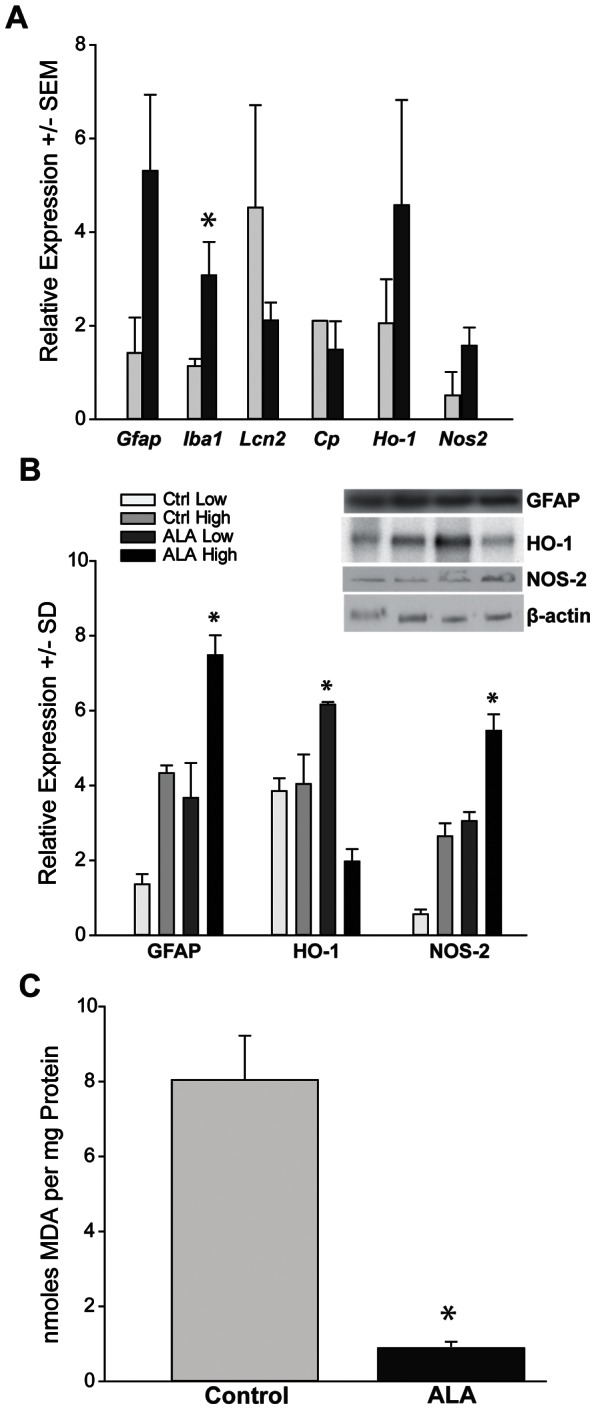
mRNA and protein changes in 12-month DBA mice fed dietary ALA from weaning (prevention experiment). **A**. Retinal analysis showed significantly more Iba1 mRNA in ALA versus control (*p = 0.01). Clear trends toward increased mRNA after ALA treatment for Gfap, Ho-1 and Nos2 were not statistically significant; by t-test, p = 0.14, p = 0.16, p = 0.08, respectively. Decreased expression for Lcn2 and Cp after ALA treatment were also not significant; by t-test, p = 0.16, p = 0.27, respectively. (n = 6 per group) **B**. Retinal protein measures show significant GFAP upregulation in retinas from the high IOP ALA treatment group (*p = 0.008); there were stable levels of HO-1 in control (low and high IOP groups), but significant elevation in the low IOP ALA treatment group (p = 0.04). NOS-2 levels were significantly higher in the high IOP ALA treatment group (p = 0.01). (n = 6 per group) **C**. Lipid peroxidation measured in pooled retina from control and ALA treated DBA mice. The protocol used measures nanomoles of MDA and 4-hydroxyalkenals. There was significantly less lipid peroxidation in ALA treated retina (*p = 0.006) (n = 8 per group).

We monitored mRNA and protein in the intervention experiment similarly to that undertaken for the prevention and baseline DBA measures. *Gfap*, *Iba1,* and *Ho-1* mRNA was higher in the ALA treatment group compared to control while *Lcn2* mRNA levels were highest in control (Figure 9). Of these changes, only the *Iba1* increase was statistically significant. Iba1 is the microglial-specific Ca^++^ binding protein; its upregulation in ALA treated mice suggests greater microglial number or activation [50] in ALA treated retina. *Lcn2* has been shown to be upregulated in DBA retina with age and increased IOP [51]. *Lcn2* levels were elevated in both ALA and control in the prevention experiment (compared to the intervention experiment); however, its slightly higher expression in control retina (prevention experiment) versus ALA treated is not significant (p = 0.13). Given the role of *Lcn2* in acute inflammatory processes, its variable elevation in the prevention experiment could suggest an element of immune regulation comes into play in the 2 month period that separates the ages of mice in the intervention (10 months) versus the prevention (12 months) experiments. *Cp* levels were unchanged between control and ALA treatment. *Nos2* levels were highest in the ALA group, in direct contrast with the findings from the intervention experiment, although the differences between groups were not statistically significant (Figure 9A). Table 4 shows a comparison of the mRNA and protein findings for the intervention and prevention experiments.

**Table 4:** Comparison of mRNA and Protein Findings for ALA intervention and prevention studies.

	mRNA			Protein	
	ALA-60 (10 m)	ALA-100 (12 m)		ALA-60 (10 m)	ALA-100 (12 m)
Cp	↑*	NC	CP	NC	NM
Gfap	NC	↑	GFAP	↑*	↑*
Ho-1	↑*	↑	HO-1	↑*	↑*
Nos2	↓*	↑	NOS2	↓*	↑*
Iba1	NC	↑*	RAGE	NC	NM
Lcn2	NC	↓			

Arrows with asterisks indicate direction of expression compared to the control group. Arrows without asterisks indicate expression trend. ALA-60 refers to mice in the intervention experiment that received 60 mg/kg/bw/day of ALA; ALA-100 refers to the prevention mice who received 100 mg/kd/bw/day of ALA. Age of mice is in parentheses. NC = no change; NM = not measured.

Similarly to the ALA group in the intervention experiment, HO-1 protein levels were increased in the ALA group for the prevention experiment (Figure 9B), though only for ALA mice with low IOP exposure. Data from mice in the prevention experiment are separated out by IOP group only when the results by group were statistically different. The high IOP group of ALA treatment retina had significantly less HO-1 than either control group (p = 0.004). These low HO-1 levels confined to the ALA group with high IOP suggests that oxidative stress response might benefit from IOP decrease. Unlike the intervention experiment, GFAP and NOS-2 protein levels in the prevention experiment were significantly increased in the high IOP group of ALA treatment retina (p = 0.008 and p = 0.01, respectively) (Figure 9B and Table 4). Our immunohistochemistry data (Figure 7) suggests that astrocytes and Müller glia (identified by GLAST antibody labeling) are likely not responsible for increased NOS-2 levels.

### Lipid Peroxidation

Important indices for oxidative stress include the presence of changes to cellular targets of oxidative stress, such as lipid, in the retina. We measured lipid peroxidation in control and ALA treated whole retina, finding significantly fewer nanomoles of malondialdehyde (MDA) and 4-hydroxyalkenal per mg of protein in the ALA treatment group (*p = 0.013), Figure 9C.

### a-Lipoic Acid Treatment Preserves RGC numbers and RGC Axons

**Figure 10:**
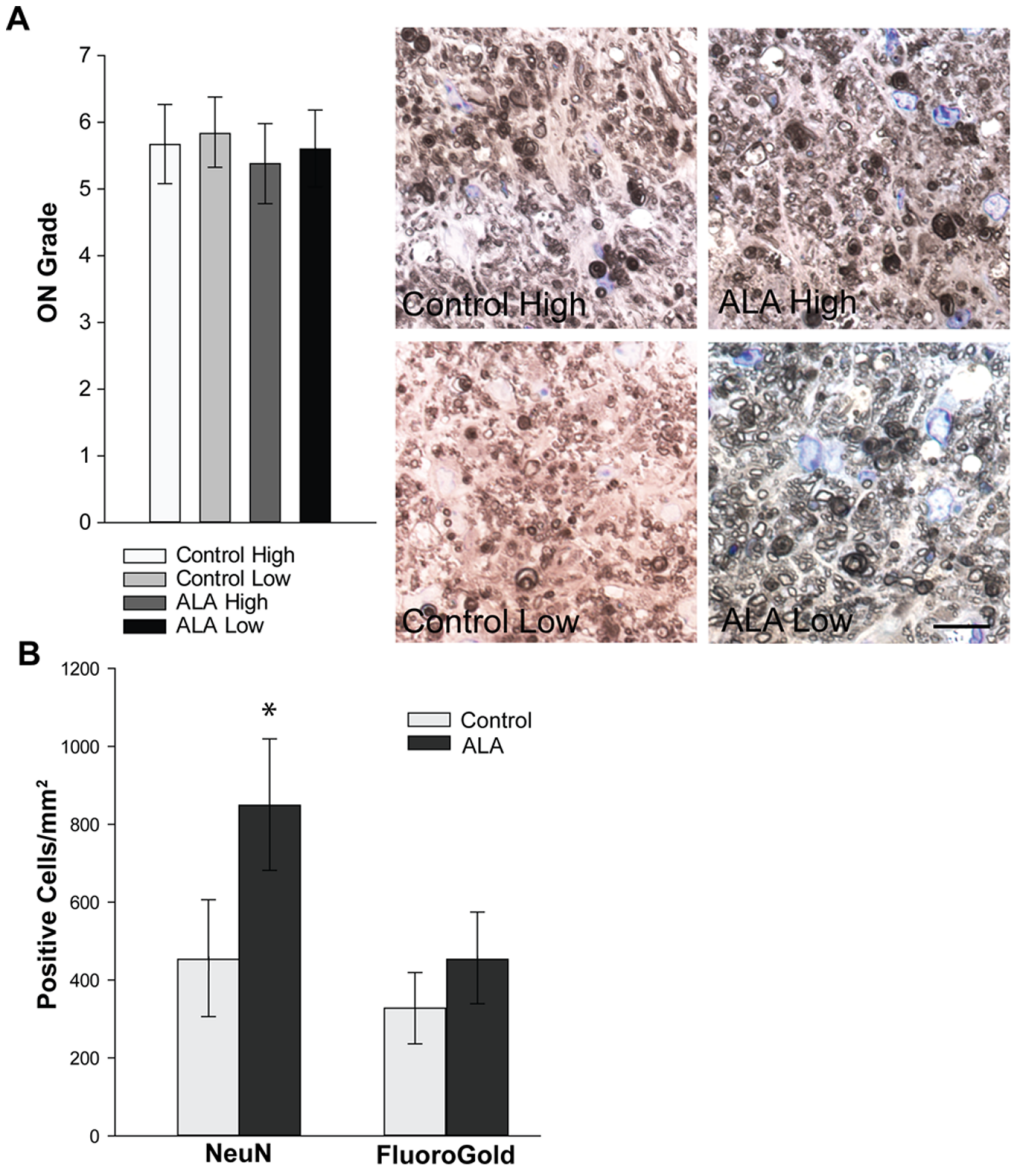
Axon degeneration and RGC number in 12-month DBA mice fed dietary ALA from weaning (prevention experiment). **A**. Optic nerves from mice in the prevention experiment were graded for level of degeneration; 0 on the scale is no degeneration; 10 is 100% degeneration (see Methods). Though average level of degeneration is lower in the ALA treatment groups regardless of IOP, there is no significant difference in ON grade among groups (t-test, p = 0.31; n = 33 per group). The example optic nerve sections shown here, stained for *p*-phenylenediamine, are all grade 7, or 70 percent degenerated. Scale bar = 10 µm. **B**. Retinas analyzed for NeuN+ and FluoroGold+ cells in the GCL and corrected for displaced amacrine cells (see Methods) show significantly higher density of NeuN+ cells in the ALA treated retinas (*p = 0.02); FG+ cells in ALA treated retinas were not statistically different from control (p = 0.13) (n = 19 for the ALA group; n = 17 for the control group). The number of NeuN-positive cells in control retinas was 9028.1 ± 2975.2 compared to 17044.2 ± 3352.7 in ALA treated retinas (p<0.018). The number of FG-positive cells in control retinas was 5831.4 ± 1679.2 compared to 9091.5 ± 2125.4 in ALA treated retinas (p = 0.11).

ALA treatment had little appreciable effect on optic nerve health in the prevention experiment, with optic nerve degeneration grades slightly lower, but not statistically so, in the ALA treatment groups, regardless of IOP (p = 0.31; Figure 10A). The optic nerve grade represents the level of degeneration (0 = no degeneration; 10 = 100 percent degeneration). Examples of optic nerves from each treatment and IOP group with a grade of 7 (70 percent degenerated) are shown (Figure 10A). As in the intervention study, we retrogradely labeled RGCs with FluoroGold and also immunolabeled the retinas with NeuN. Unbiased stereological counts of those retinas, corrected for displaced amacrine cells, showed greater numbers of NeuN+ cells in the GCL of ALA treated retina (Figure 10B). Control retinas have 36 percent fewer FG+ cells (not statistically different) and 47 percent fewer NeuN+ cells than ALA treated retinas. The prevention experiment mice were 12 months of age and show significant decrease of cell density when compared to the 10-month-old retinas from the intervention experiment (compare Figures 8D and 10B). Nevertheless, the ALA treatment did result in greater RGC preservation (p = 0.02).

### Visual Activity Testing

Fundamental to the motivation for protecting RGCs is the goal of maintaining visual function. In order to test visual function in the control and ALA treated mice in the prevention experiment, dark-adapted mice were exposed to a 450-lux overhead light while sitting in either a dark box or striped box for ten minutes (see Methods). The mouse brains were then processed for c-fos immunochemistry and cells in V1 visual cortex were counted. The end point of the study, 12 months of age, was late enough in the course of disease progression that equivalent levels of c-fos-positive cells were observed in both control and ALA mice. The c-fos+ cell numbers observed were lower than the negative control, suggesting very little to no visual signal was being processed in V1 of either control or ALA mice (Figure S3).

### Mechanism of ALA Protection

**Figure 11:**
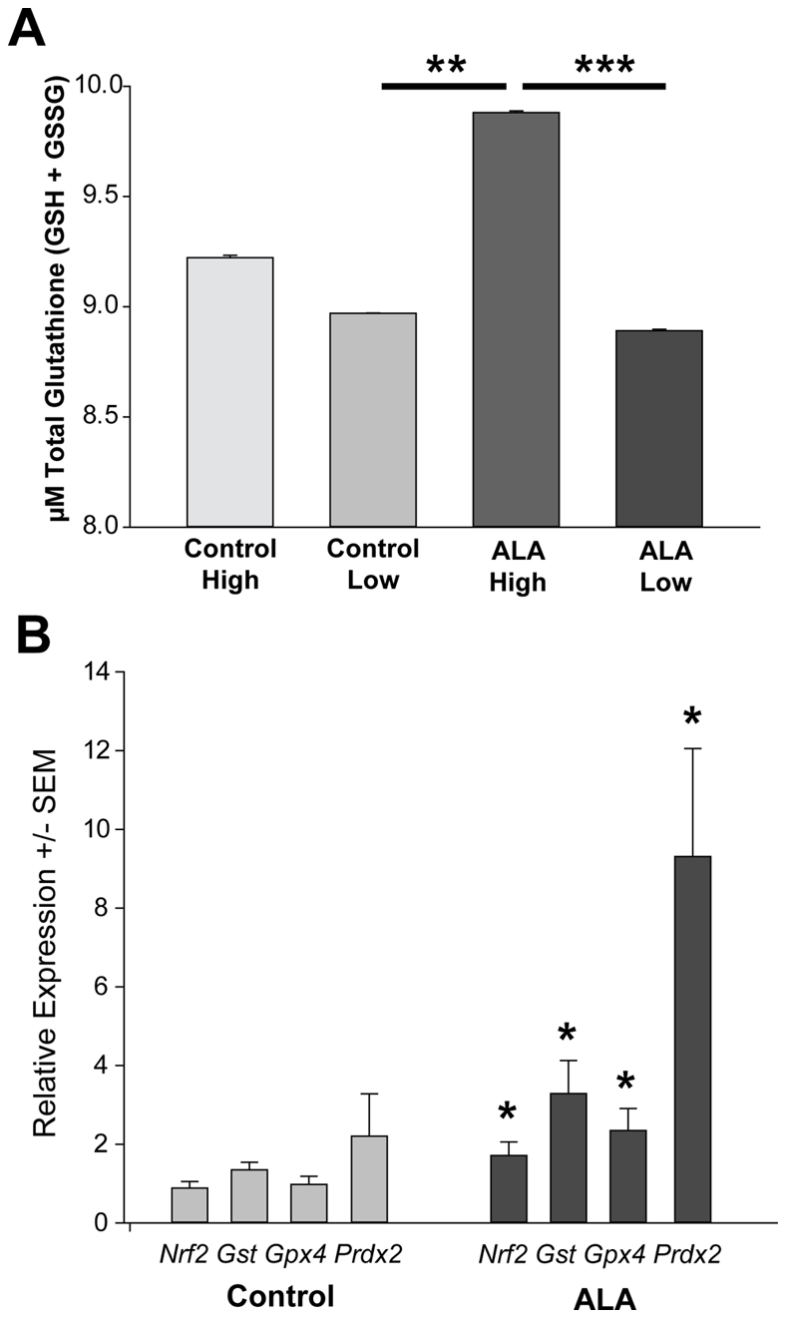
ALA treatment increased antioxidant response in DBA mice. **A**. Glutathione (in its reduced GSH and oxidized GSSG forms), a major antioxidant in the CNS, was measured in the retinas of ALA treated mice. ALA treated retinas from mice with the highest IOP have significantly more glutathione than either control or ALA treated mice with low IOP (**p = 0.01 and ***p = 0.006, respectively) (n = 5 per group). **B**. Relative expression of the transcription factor Nrf2 and its downstream target antioxidant genes glutathione-S-transferase (GST), glutathione peroxidase 4 (GPx4), and peroxiredoxin 2 (Prdx2) showed significant increases in ALA treated retina compared to control (*p<0.05) (n = 5 per group). Expression levels were normalized to a panel of housekeeping genes (see Methods).

ALA could exert a protective effect on RGCs in various ways, so we undertook experiments to ascertain changes in levels of antioxidant related genes and glutathione in control and ALA treated mice. Whole retina from ALA-treated mice with high IOP had significantly greater glutathione than both ALA-treated (low IOP) retina and control (low IOP) retina (Figure 11A). The Nrf2 transcription factor, which binds to the antioxidant response element (ARE) to regulate expression of several antioxidant-related genes, gets released from its regulatory partner Keap1 in conditions of oxidative stress [52]. We assayed *Nrf2* and Nrf2-ARE target gene mRNA levels in order to determine if this regulatory system could be implicated in oxidative stress reduction in ALA-treated mice. Nrf2, glutathione-S-transferase, glutathione peroxidase 4 and peroxiredoxin 2 were all upregulated in the ALA treated retina (Figure 11B), suggesting that these components of the cellular antioxidant defense increased as a result of ALA treatment.

Oxidative stress can exacerbate age-related changes in structures such as cornea [53], and the age and IOP-related corneal changes of DBA/2J mice have been well documented [39], [54]. An analysis of control and ALA treated corneas in the mice used for the glutathione and *Nrf2* mRNA analyses demonstrated a significant decrease in the incidence of corneal damage; in this case, corneal occlusions, in the ALA treated mice (Table 5). Figure S4 shows a photomicrograph of a mouse cornea with corneal occlusions.

**Table 5:** Incidence of Corneal Occlusions in eyes from the ALA prevention study.

	Control	ALA
Clear Cornea	0	20
1 to 5 Occlusions	15	2
5+ Occlusions	5	0

## Discussion

Oxidative stress is an underlying observation of many neurodegenerative disorders. In this study, we characterized the early oxidative stress processes and the response of the innate antioxidant pathways in the DBA retina during glaucoma progression. We found increases in lipid peroxidation, protein nitration and oxidation of nucleic acids that correlate with the decline in RGC function. Concomitant with these changes were increases in antioxidant proteins such as CP and HO-1, likely a natural protective mechanism to manage the mounting oxidative stress load. RAGE, a contributor to antioxidant depletion, was also increased. These data collectively indicated that the DBA retina is susceptible to particular oxidative stress-related decline. Based on this analysis, we delivered the dietary antioxidant α-lipoic acid to the mice with a goal to intervene or prevent glaucoma development by increasing antioxidant availability. Our data demonstrate that ALA decreases oxidative stress and limits protein nitration, DNA oxidation, and lipid peroxidation. We have identified changes in key antioxidant enzymes that may shed light on the mechanism of the protective effects of ALA. These data are particularly exciting as they show the potential for a dietary approach to increase innate antioxidant capacity that leads to maintenance of RGCs.

Our data showed evidence of decreased byproducts of oxidative stress, no doubt as a consequence of the availability of ALA. In addition to scavenging reactive oxygen species [27], regenerating endogenous antioxidants and repairing oxidative damage [28], ALA may itself contribute to Nrf2 target gene induction through its structural similarity to dithiolethiones, compounds that can relieve the regulatory binding of Nrf2 by Keap1, allowing Nrf2 to translocate to the nucleus [27]. ALA relief of Nrf2 repression would explain the increase in Nrf2 antioxidant enzymes with ALA treatment, including the increased glutathione levels in the ALA prevention group compared to control. The modifier and catalytic subunits of the glutamate-cysteine ligase, enzymes in the biosynthetic pathway of the antioxidant glutathione, are both proteins regulated by the Nrf2-ARE. HO-1 mRNA and protein were consistently elevated in the intervention and prevention ALA study groups. HO-1 is regulated by the Nrf2-ARE; hence, its upregulation is consistent with the increase in Nrf2 and Nrf2 target genes observed in the ALA prevention retinas. These results, in addition to the mRNA findings, underscore the improved availability of antioxidants that occurred with ALA treatment. Systemic antioxidant treatment in humans has been shown to decrease the health benefits of physical exercise [55]. One explanation for the detrimental effect of antioxidant treatment is that the exogenous antioxidants short-circuit the feedback mechanisms for initiating endogenous antioxidant response, feedback that includes accumulation of oxidative stress [56]. Conversely, our data show that chronic antioxidant treatment had no negative effect on endogenous antioxidant response in the mouse retina.

Nrf2-ARE activation in astrocytes can protect neurons from oxidative insult [16], [57]. In this study, dietary ALA would have been available to all retinal cells, and therefore capable of increasing antioxidants globally. Though we have evidence that Nrf2-ARE activation was increased in ALA treated retinas, we do not know if activation occurred primarily in glia, neurons or both. It is interesting to consider if targeted upregulation of antioxidants in glia, for example, would have a greater neuroprotective effect in this model. ALA may also contribute to the overall health of RGCs and the retina by dampening inflammatory processes [58] and enhancing mitochondrial function. Iba1 mRNA levels, which indicate either microglial proliferation/infiltration or increased activation of the existing microglial population [50], [59], were unchanged in the intervention but increased in the prevention experiment. This result suggests that chronic ALA treatment had little effect on microglia with 4 months of treatment, but possibly increased microglial activation with 11 months of treatment. Alternatively, the two additional months of aging experienced by mice in the prevention experiment might have contributed to the microglial activation. The Nrf2 upregulation that occurred with the prevention experiment might have been expected to decrease inflammation because Nrf2 activation inhibits NFκB [60], the redox-sensitive transcription factor that can be inhibited by antioxidants [58], including ALA. It is possible that Iba1+ microglia were activated through some glaucoma-related mechanism and the ALA-related Nrf2 upregulation could counteract the downstream effects of microglial activation but not the activation itself. This possibility merits further investigation.

As byproducts of cellular processes, ROS are signals as much as they are reactive molecules whose elimination can benefit cell survival. Nitric oxide can be pro- or anti-inflammatory, depending on the site and amount released [61]. For example, nitric oxide can inhibit NFκB activation, thereby limiting inflammatory response. NOS-2 levels are low compared to control in our intervention experiment, though NOS-2 levels are increased in the prevention study. NOS-2 activity has generally been thought to be negative in the context of glaucoma since NOS-2 induced by ocular hypertension can lead to protein nitration [62]. Nrf2 can repress NOS-2 activity [60], yet higher Nrf2 levels were not sufficient to keep NOS-2 levels in check in the prevention experiment. This might not have significant implications for glaucoma pathology since it has been shown that either knocking out NOS-2 on the DBA/2J background or inhibiting NOS-2 with aminoguanidine conferred no protection of RGCs or their axons [63]. Nitric oxide (NO) and ALA, both capable of playing an anti-inflammatory role, are nevertheless pitted against each other, with ALA eliminating NO in the intervention experiment via NOS-2 downregulation while having little effect in the prevention experiment. These observations underscore the complex interactions of signaling molecules that are also implicated in oxidative stress; more antioxidants is not necessarily better. Interestingly, antioxidants have been observed to facilitate neurodegeneration by inhibiting basal autophagy [64]. These data were obtained in a model of polyglutamine disease, but suggest that there is an important balance that much be achieved between eliminating destructive reactive oxygen species and maintaining functional cell signaling. Relatedly, there is evidence that antioxidants might need to be tailored to counter the specific source of reactive oxygen species for maximal therapeutic benefit [65]. Part of that tailoring could include providing antioxidants or the capability to generate antioxidants to specific cell types as opposed to global treatment as was undertaken here.

One motivation for antioxidant therapy is an assumption that endogenous antioxidant mechanisms are inadequate to counter the disease-related oxidative stress. Similar to diabetic retinopathy [66], high antioxidant levels have been observed in neurodegenerative diseases like multiple sclerosis at the same time there is evidence of significant oxidative stress [67]. Though ALA treatment led to lower levels of lipid peroxidation and higher antioxidants in the intervention study, these changes did not fully prevent glaucoma-related declines in RGC number and function. A number of antioxidant genes attain normal levels in the prevention study. Again, limiting oxidative stress, as in either the intervention or the prevention study, can impact RGC structure and function but does not reverse glaucoma pathogenesis. The impact of ALA treatment on the various outcome measures monitored in the two studies indicates the magnitude of the contribution oxidative stress makes to the pathogenesis of glaucoma. This is important information for therapy; oxidative stress does indeed contribute in a meaningful way to glaucoma pathology and should thus be incorporated into treatment plans.

A critical piece of evidence for the preservation of RGC structure would be a demonstration of function as well. We chose to measure c-fos expression in the visual cortex for mice in the prevention experiment, but observed no evidence of visual activity. The lack of c-fos expression in V1, in retrospect, was unsurprising given the recent publication demonstrating no visual acuity in 12-month-old DBA mice [68]. Our method, assessing c-fos levels in V1, precluded a longitudinal analysis of vision, but it will be important in the future to monitor visual acuity throughout the course of disease progression.

In glaucoma, there is growing realization that an increase in IOP sets a pathological process in motion that persists beyond any initial cellular injury. This fact has significance for the therapeutic options of glaucoma patients who do not respond to IOP lowering treatment. The mechanism of such a phenomenon is not yet clear, though there is precedent for the concept in studies of diabetic retinopathy where mitochondria have been implicated in the “metabolic memory” that persists during good glycemic control. In diabetic retinopathy and in normal aging, ALA is reported to alleviate mitochondrial metabolic abnormalities, likely through superoxide radical scavenging [30]. Likewise in our previous studies of glaucoma, we have observed metabolic deficiency in the DBA/2J optic nerve that coincides with an early period of degeneration [69]. In the future, it will be important to uncover the mechanisms of ALA action and how RGC function is supported even in the presence of high IOP. Such studies are important in order to design more potent therapeutic strategies and co-therapies but also to inform us of the underlying forces that drive glaucoma progression when IOP lowering therapies fail. In the short term, these preclinical data provide a provocative rationale for testing an antioxidant regimen in glaucoma patients.

## Supporting Information

Figure S1 **IOP exposure for the glaucoma prevention experiment.** IOP exposure, expressed as mmHg-Days, was calculated by multiplying the measured IOP by the number of days at that IOP and then summing the values. There was no difference in IOP exposure between control and ALA treatments within the low or high IOP category, but the average difference across low and high IOP categories (≥600 mmHg-Days) is significant (*p = 0.001). All mRNA and protein analysis for the prevention experiment was done by IOP level in the control and ALA treatment groups, but was only presented by IOP level when the results were statistically different.
(DOCX)

Figure S2 **Immunolocalization for βIII-tubulin in retinal whole mounts from control DBA mice showed smaller RGC somas and thinner axons in both the peripheral and central retina when compared to ALA treated DBA mice.** Images were captured from similar areas of their respective retinas (green). Scale bar = 10 µm.
(DOCX)

Figure S3 **Cells immunolabeled for c-fos in V1 visual cortex were counted.** The negative control was a 6-month-old DBA/2J mouse exposed to a black box and the positive control was a 6-month-old DBA/2J mouse exposed to a striped box. Both ALA treated and control 12-month DBA/2J mice had marginal c-fos cell numbers in V1, below the level of the negative control. The positive control has significantly greater c-fos expression than the negative control and each of the ALA and control groups (*p<0.05); n = 6 for each condition.
(DOCX)

Figure S4 **Corneal appearance.** The subset of mice used for FluoroGold and NeuN analysis were scored for corneal occlusions. A sample image of several corneal occlusions in one eye is shown. Twenty of the ALA treated mice were completely free of corneal occlusions (clear) whereas all of the control mice had at least one and some more than five occlusions.
(DOCX)
