# Supplementary material for: α-Lipoic Acid Antioxidant Treatment Limits Glaucoma-Related Retinal Ganglion Cell Death and Dysfunction
Source: PLoS One. 2013 Jun 5;8(6):e65389. doi: 10.1371/journal.pone.0065389 (PMC3673940; doi:10.1371/journal.pone.0065389)
Supplement: Figure S1 — IOP exposure for the glaucoma prevention experiment. IOP exposure, expressed as mmHg-Days, was calculated by multiplying the measured IOP by the number of days at that IOP and then summing the values. There was no difference in IOP exposure between control and ALA treatments within the low or high IOP category, but the average difference across low and high IOP categories (≥600 mmHg-Days) is significant (*p = 0.001). All mRNA and protein analysis for the prevention experiment was done by IOP level in the control and ALA treatment groups, but was only presented by IOP level when the results were statistically different. (DOCX) [file pone.0065389.s001.docx]

**Supplementary Information**

**Figure S1**


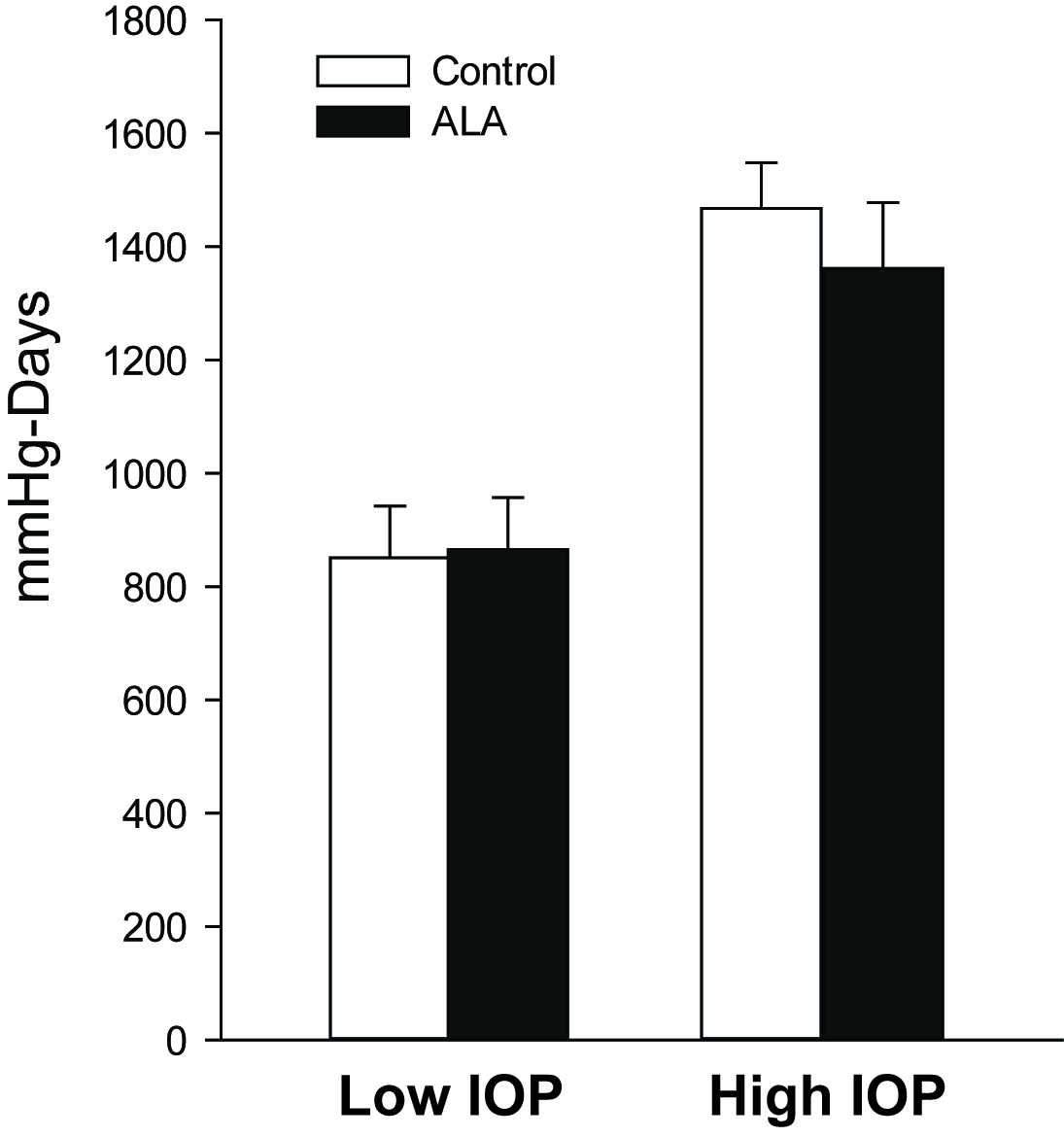


**Suppl. Figure 1**. **IOP exposure for the glaucoma prevention experiment.** IOP exposure, expressed as mmHg-Days, was calculated by multiplying the measured IOP by the number of days at that IOP and then summing the values. There was no difference in IOP exposure between control and ALA treatments within the low or high IOP category, but the average difference across low and high IOP categories (≥600mmHg-Days) is significant (*p=0.001). All mRNA and protein analysis for the prevention experiment was done by IOP level in the control and ALA treatment groups, but was only presented by IOP level when the results were statistically different.
