# Supplementary material for: α-Lipoic Acid Antioxidant Treatment Limits Glaucoma-Related Retinal Ganglion Cell Death and Dysfunction
Source: PLoS One. 2013 Jun 5;8(6):e65389. doi: 10.1371/journal.pone.0065389 (PMC3673940; doi:10.1371/journal.pone.0065389)
Supplement: Figure S2 — Immunolocalization for βIII-tubulin in retinal whole mounts from control DBA mice showed smaller RGC somas and thinner axons in both the peripheral and central retina when compared to ALA treated DBA mice. Images were captured from similar areas of their respective retinas (green). Scale bar = 10 µm. (DOCX) [file pone.0065389.s002.docx]

**Supplementary Information**

**Figure S2**


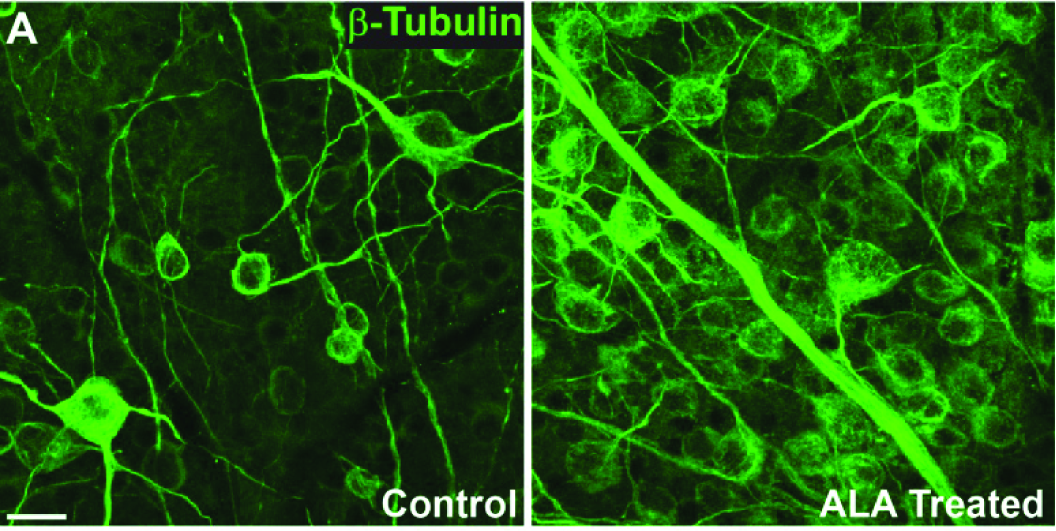


Immunolocalization for βIII-tubulin in retinal whole mounts from control DBA mice showed smaller RGC somas and thinner axons in both the peripheral and central retina when compared to ALA treated DBA mice. Images were captured from similar areas of their respective retinas (green). Scale bar=10µm.
