# Supplementary material for: α-Lipoic Acid Antioxidant Treatment Limits Glaucoma-Related Retinal Ganglion Cell Death and Dysfunction
Source: PLoS One. 2013 Jun 5;8(6):e65389. doi: 10.1371/journal.pone.0065389 (PMC3673940; doi:10.1371/journal.pone.0065389)
Supplement: Figure S3 — Cells immunolabeled for c-fos in V1 visual cortex were counted. The negative control was a 6-month-old DBA/2J mouse exposed to a black box and the positive control was a 6-month-old DBA/2J mouse exposed to a striped box. Both ALA treated and control 12-month DBA/2J mice had marginal c-fos cell numbers in V1, below the level of the negative control. The positive control has significantly greater c-fos expression than the negative control and each of the ALA and control groups (*p<0.05); n = 6 for each condition. (DOCX) [file pone.0065389.s003.docx]

**Supplementary Information**

**Figure S3**


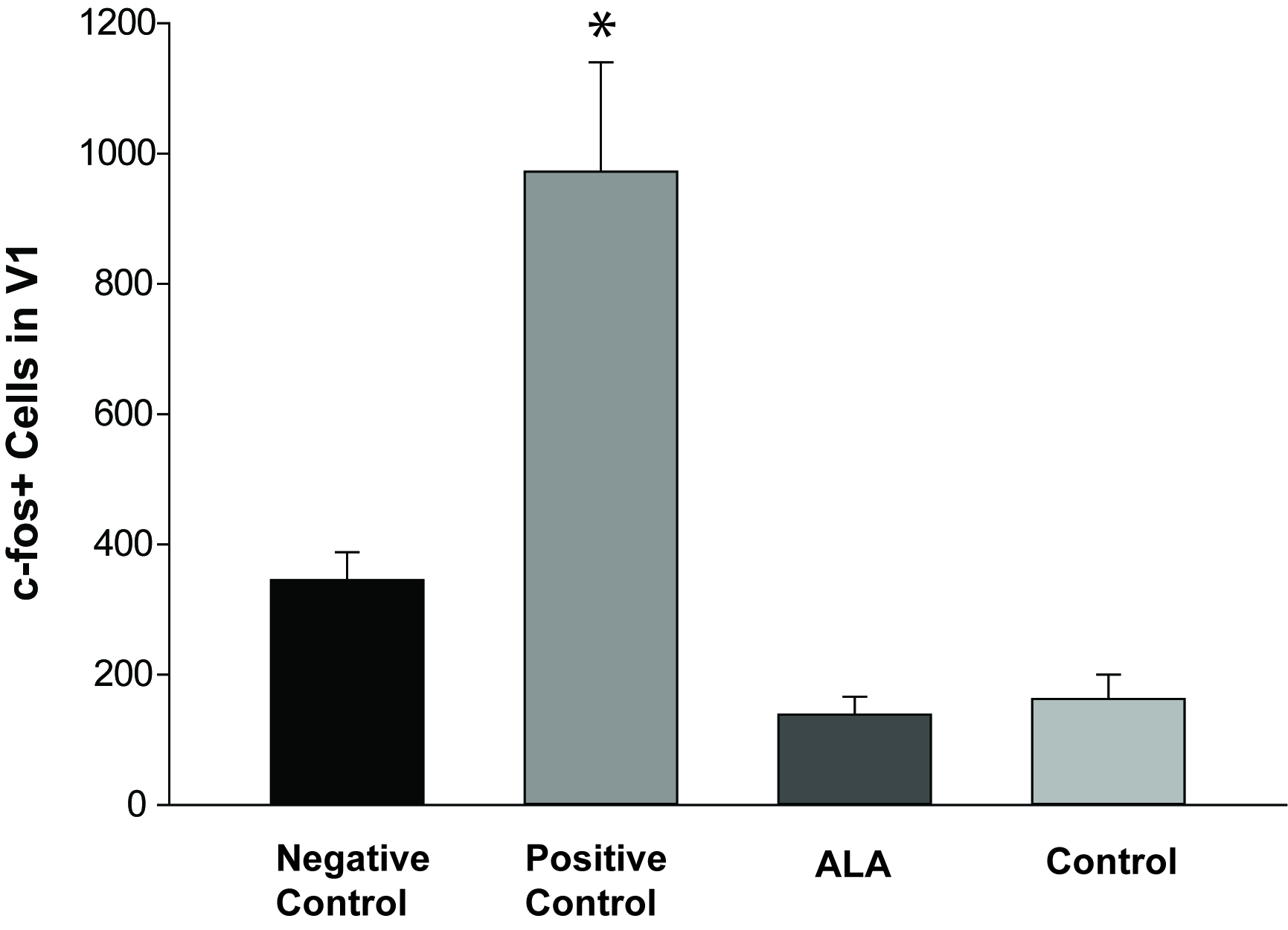


Cells immunolabeled for c-fos in V1 visual cortex were counted. The negative control was a 6-month-old DBA/2J mouse exposed to a black box and the positive control was a 6-month-old DBA/2J mouse exposed to a striped box. Both ALA treated and control 12-month DBA/2J mice had marginal c-fos cell numbers in V1, below the level of the negative control. The positive control has significantly greater c-fos expression than the negative control and each of the ALA and control groups (*p<0.05); n=6 for each condition.
