# Supplementary material for: α-Lipoic Acid Antioxidant Treatment Limits Glaucoma-Related Retinal Ganglion Cell Death and Dysfunction
Source: PLoS One. 2013 Jun 5;8(6):e65389. doi: 10.1371/journal.pone.0065389 (PMC3673940; doi:10.1371/journal.pone.0065389)
Supplement: Figure S4 — Corneal appearance. The subset of mice used for FluoroGold and NeuN analysis were scored for corneal occlusions. A sample image of several corneal occlusions in one eye is shown. Twenty of the ALA treated mice were completely free of corneal occlusions (clear) whereas all of the control mice had at least one and some more than five occlusions. (DOCX) [file pone.0065389.s004.docx]

**Supplementary Information**

**Figure S4**

**Corneal appearance**


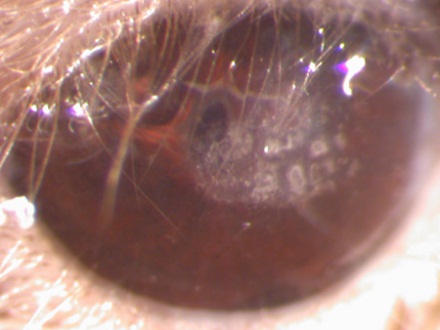


The subset of mice used for FluoroGold and NeuN analysis were scored for corneal occlusions. A sample image of several corneal occlusions in one eye is shown. Twenty of the ALA treated mice were completely free of corneal occlusions (clear) whereas all of the control mice had at least one and some more than five occlusions.

|  | **Clear** | **1-5 Occlusions** | **5+ Occlusions** |
| --- | --- | --- | --- |
| ALA | 20 | 2 | 0 |
| Control | 0 | 15 | 5 |
